# Case-management protocol for bloody diarrhea as a model to reduce the clinical impact of Shiga toxin-producing *Escherichia coli* infections. Experience from Southern Italy

**DOI:** 10.1007/s10096-019-03755-0

**Published:** 2019-11-27

**Authors:** Daniela Loconsole, Mario Giordano, Nicola Laforgia, Diletta Torres, Luisa Santangelo, Vincenza Carbone, Antonio Parisi, Michele Quarto, Gaia Scavia, Maria Chironna, Bloody Diarrhea Apulia Working Group, Luigi Nigri, Viviana Bruno, Simona Baldacci, Francesca Centrone, Anna Lisa De Robertis, Anna Morea, Daniele Casulli, Marisa Accogli, Serafina Rutigliano, Onofrio Mongelli

**Affiliations:** 1 Department of Biomedical Sciences and Human Oncology, Hygiene Unit, University of Bari Aldo Moro, P.zza G. Cesare 11, 70124 Bari, Italy; 2 Pediatric Nephrology and Dialysis Unit, Pediatric Hospital “Giovanni XXIII”, Bari, Italy; 3 Department of Biomedical Sciences and Human Oncology, Neonatal Intensive Care Unit, University of Bari Aldo Moro, Bari, Italy; 4 Istituto Zooprofilattico Sperimentale della Puglia e della Basilicata, Foggia, Italy; 5 Food Safety, Nutrition and Veterinary Public Health Department, Istituto Superiore di Sanità, Rome, Italy

**Keywords:** Shiga toxin-producing *Escherichia coli*, Bloody diarrhea, Early volume expansion, Hemolytic uremic syndrome

## Abstract

To describe an operating protocol for bloody diarrhea (BD) in a pediatric population as a rapid response to a public health threat represented by an excess of pediatric HUS cases in the Apulia region (Southern Italy) starting from 2013. The protocol was set up with the goal of correct clinical management of Shiga toxin-producing *Escherichia coli* (STEC) infections, reductions in subsequent cases of hemolytic uremic syndrome (HUS), and improved short- and long-term disease outcomes. The protocol consisted of rapid hospitalization of children with bloody diarrhea (BD), hematochemical laboratory tests every 12–24 hours, and prompt laboratory diagnosis of STEC. No antibiotics were recommended until diagnosis. Children positive for STEC infections underwent early vigorous volume expansion. In June–December 2018, 438 children with BD were hospitalized, of which 53 (12.1%) had a STEC infection. The most common serogroups were O26 (36.1%), O111 (23.0%), and O157 (14.8%). Thirty-one samples carried the *stx2* gene. Four cases evolved into HUS (7.5%), all with favorable outcome despite neurological involvement in two cases. Prompt and accurate laboratory diagnosis of STEC infections is of the utmost importance in patients with BD for correct clinical management. The strict adherence to the protocol could reduce the progression rate of STEC infections to HUS and prevents complications. Enhanced BD surveillance may help reduce cases of pediatric HUS in Southern Italy.

## Introduction

Shiga toxin-producing *Escherichia coli* (STEC) infections are a persisting public health concern. Symptoms of STEC infections often include abdominal cramps, diarrhea, and bloody diarrhea (BD). Fever and vomiting may also occur [1, 2]. The incubation period can range from 3 to 8 days, with a median of 3–4 days [2].

It is estimated that up to 10–15% of patients with a STEC infection may develop hemolytic-uraemic syndrome (HUS), with children being particularly vulnerable. HUS is characterized by microangiopathic hemolysis, platelet consumption, and multiple organ damage (particularly renal failure), with a case-fatality rate ranging from 3% to 5% [1, 2]. Overall, HUS is the most common cause of acute kidney failure requiring hospital care in young children. Neurological complications occur in 25% of HUS patients [2]. Hemoconcentration and dehydration are risk factors for more severe hematologic, renal, and neurologic involvement in HUS cases [3–9]. Generally, children with a STEC infection and BD are not hospitalized until HUS development [10], thus missing the opportunity to mitigate the progression of the disease through early volume expansion to reduce organ damage [3, 11]. Such early interventions have positive effects on both short- and long-term disease outcomes [12].

STEC are considered pathogens of top priority in the European Union (EU), and surveillance of STEC infections is mandatory [13]. In Italy, reports of STEC infections are mainly comprised of cases of HUS in children (< 15 years) reported via the Italian National Registry of HUS [14]. HUS is considered a robust sentinel event revealing the circulation of STEC in the general population. Surveillance data are published annually and showed a progressive upward trend in HUS cases over the last decade, with an average of 55.4 cases/year in the 2009–2018 period [13].

In recent years, cases of HUS increased dramatically in number and severity in the Apulia region of Southern Italy. In summer 2013, a community-wide outbreak of HUS caused by stx2-producing *E. coli* O26 involved 20 children, with two of them reporting severe neurological sequelae [15]. In 2017, a higher-than expected number of HUS cases (*N* = 18) was registered in the summer months, involving both indigenous patients as well as patients not residing in Apulia, with two deaths. Between January and the second week of June 2018, a further four cases of HUS occurred, with one death (a 13-month-old toddler) (data from Regional Epidemiological Observatory, not published). Multiple STEC serogroups (O26, O45, O111, O121, O145, O157) have been associated with such cases (data from Regional Epidemiological Observatory, not published). Following these events, the Regional Health Authorities together with a multidisciplinary team of healthcare professionals set up an enhanced laboratory-based surveillance protocol for the pediatric population developing BD, with an “admit-all” approach aimed at reducing the health burden of STEC infections and better characterizing their epidemiology in the Apulia region. Here, we describe the results of this prospective surveillance from June to December 2018.

## Materials and methods

The Apulia region in Southern Italy has a population of over four million people, 581,432 of whom are children below the age of 15 (Source demographic data: ISTAT, 2018; http://demo.istat.it/pop2018/index.html). Throughout the region, there are 24 hospitals with a pediatric unit. The population under surveillance included children < 15 years old, presenting to a primary care pediatrician or to public pediatric outpatient services in the Apulia region. Cases under study were defined as children presenting and/or reporting BD during the previous 2 weeks, defined as the presence of any amount of blood in the loose stool at presentation or as reported by the parents. Patients with BD for longer than 2 weeks or with a known noninfectious gastrointestinal disease were excluded. A multidisciplinary team comprised of public health professionals, pediatricians, and microbiologists set up a regional operating protocol based on rapid hospitalization and STEC laboratory testing of stool samples of children with BD. All procedures performed were in accordance with the 1964 Helsinki declaration and its later amendments. Ethical approval was obtained from the Institutional Review Board at the Apulian Regional Observatory for Epidemiology. Informed written consent was obtained from all legal guardians of the children who provided the specimens.

### Operating protocol

**Fig. 1:**
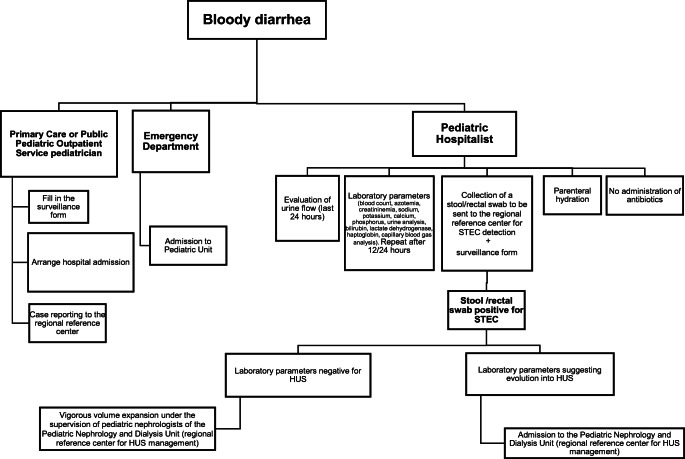
Flow chart for the management of bloody diarrhea, Southern Italy, 2018

The management of each BD case was conducted according to the protocol illustrated in the flow chart shown in Fig. 1, and described as follows. In the case of a child with BD, the pediatrician should arrange immediate hospital admission at the nearest hospital with a pediatric unit, fill in the surveillance form with the patient’s personal data, symptoms, and antibiotic therapy, and report the case, through an e-mail alert, to the Hygiene Unit of the Azienda Ospedaliero-Universitaria Policlinico university hospital, Bari (Italy), which is the Regional Reference Center. Children presenting directly at a hospital emergency department (ED) with BD should be admitted to the nearest pediatric unit. According to the protocol, the pediatric hospitalist should evaluate the continued urine flow from the previous 24 h and assess the following laboratory parameters at admission and again after 12–24 h: blood count, azotemia, creatininemia, sodium, potassium, calcium, phosphorus, urine analysis, bilirubin, lactate dehydrogenase, haptoglobin, and capillary blood gas analysis. Furthermore, at the admission, there should be a prompt collection of a stool sample or a rectal swab for the laboratory STEC test. Antibiotics administration is not recommended until diagnosis. Parenteral hydration is recommended for each child with BD. According to the operating protocol, the results of the laboratory test performed on the stool sample should be communicated to the pediatric hospitalists within 24 h of the arrival of fecal samples. The diagnosis of HUS was suspected in presence of clinical signs of active thrombotic microangiopathy (low platelet count, hemolysis, and renal failure). In cases with STEC infection but where the laboratory parameters are not suggestive of HUS, early vigorous volume expansion should be carried out under the supervision of a pediatric nephrologist of the Pediatric Nephrology and Dialysis Unit of the Giovanni XXIII Hospital, Bari (the Regional Reference Center for HUS management). For all cases, the infusion protocol is 10–15 ml/kg/h of isotonic solution [12]. Careful monitoring of continued urine flow is mandatory. In cases with STEC infection that the laboratory parameters suggest is evolving into HUS, the patient should be promptly admitted to the Pediatric Nephrology and Dialysis Unit of the Giovanni XXIII Hospital, Bari, for appropriate clinical and therapeutic management. In cases with no STEC infection, reducing fluid administration is left entirely to the pediatric hospitalists.

### Laboratory diagnosis of STEC infection

The tests for STEC detection and identification were performed at the Laboratory of Molecular Epidemiology and Public Health of the Hygiene Unit of the Azienda Ospedaliero-Universitaria Policlinico university hospital, Bari (Italy). Stool samples or rectal swabs from cases under surveillance were subjected to real-time PCR assay for the molecular detection of STEC and other common agents of BD, namely, *Campylobacter coli/jejuni*, *Salmonella* spp, *Shigella* spp*/E. coli enteroinvasive (EIEC)*, *Yersinia enterocolitica*, and *Clostridium difficile.* Total nucleic acid was extracted with the MagNAPure LC-automated extraction system (Roche Diagnostics, Milan, Italy). A commercial real-time PCR kit was used for detection of the stx1/stx2-coding genes (FTD Bacterial Gastroenteritis, Fast track Diagnostics, Luxembourg). Samples that tested positive for STEC infection were subsequently submitted to serogroup identification through a real-time PCR commercial test (STEC Identification LyoKit, BIOTECON Diagnostics GmbH, Potsdam, Germany) and to a real-time PCR test which discriminated between the *stx1* and *stx2* genes (PATHfinder *E. coli* VTEC stx1-stx2 & eae-IAC Duplex Assay, Generon, San Prospero (MO), Italy).

### Data analysis

Data analysis was performed on the information collected from the surveillance forms. Continuous data are reported as medians with the interquartile range (IQR), except where otherwise specified. Statistical analyses were performed with STATA 12.0 software. The 95% confidence intervals are reported for proportions. The significance of the difference in categorical variables between pathogen-positive STEC patients and pathogen-positive non-STEC patients was determined with a chi^2^-test. For continuous variables, the significance of the difference between the group medians was determined with a two-tailed Mann-Whitney U Test.

## Results

Between June and December 2018, 438 cases of BD, corresponding to a mean estimated incidence of 129.1 cases per 100,000 resident population (0–15 years), were detected and hospitalized in 23/24 Pediatric Units of the Apulia region hospitals. Two hundred and thirty-seven patients were male (54.1%) and the median age was 3 years (IQR, 1.5–7 years; range, 0–16 years). Other demographic and clinical characteristics of the children are reported in Table 1. Two hundred and eighty-nine cases (65.8%) tested positive for the presence of at least one pathogen (hereafter denoted as positive cases). STEC was detected in 53 cases (12.1%). Stools/rectal swabs from 236 patients (53.9%) contained a non-STEC bacterial pathogen (*Campylobacter coli/jejuni*, *n* = 156; *Salmonella* spp, *n* = 51; *Shigella* spp*/E. coli enteroinvasive (EIEC)*, *n* = 4; *Yersinia enterocolitica, n* = 2; *Clostridium difficile, n* = 16; coinfections, *n* = 7)*.* Compared with the positive non-STEC children, children infected with STEC were younger (2.67 years vs. 4.2 years, *p* = 0.07), although this difference was not statistically significant (Table 2). We also analyzed differences in the clinical characteristics of positive STEC children and positive non-STEC children. As a group, positive non-STEC children were more likely to have a fever at presentation and a higher median number of stools in the previous 24 h than children infected with STEC (*p* < 0.05). The average duration of symptoms at hospital admission (measured as the time from the onset of BD) was 19.2 h for positive STEC children and 18.9 h for positive non-STEC children.

**Table 1:** Demographic and clinical characteristics of the pediatric study population, Southern Italy 2018

Characteristics	Cases of BD (n = 438)
	Number (%, CI 95%)
Sex	
Male	237 (54.1%, 49.3–58.8)
Female	201 (45.9%, 41.1–50.7)
Median age	3 (IQR: 1.5–7)
Resident	413 (94.3%, 91.7–96.3)
Symptoms	
Fever	234 (53.4%, 48.6–58.2)
Vomiting	107 (24.4%, 20.4–28.7)
Relatives with diarrhea	57 (13%, 10.0–16.5)
Antibiotic use	75 (17%, 13.7–20.9)
No. of stools in previous 24 h (median)	4 (IQR: 3–7)

**Table 2:** Demographic and clinical characteristics of positive STEC children and positive non-STEC children, Southern Italy 2018

Characteristics	Positive STEC children (*n* = 53)	Positive non-STEC children^a^ (n = 236)	*p value*
	Number (%, CI 95%)	Number (%, CI 95%)	
Sex			
Male	27 (50.9%, 36.8–64.9)	129 (54.7%, 48.1–61.1)	0.62
Female	26 (49.1%, 35.0–63.1)	107 (45.3%, 38.9–51.9)	0.62
Median age	2.67 (IQR: 1–7)	4.2 (IQR: 1.8–8)	0.07
Resident	49 (92.5%, 81.8–97.9)	221 (93.6%, 89.7–96.4)	0.75
Symptoms			
Fever	19 (35.8%, 23.1–50.2)	174 (73.7%, 67.6–79.2)	< 0.001
Vomiting	13 (24.5%, 13.7–38.3)	63 (26.7%, 21.1–32.8)	0.538
Relatives with diarrhea	5 (9.4%, 3.1–20.6)	31 (13.1%, 9.1–18.1)	0.64
Antibiotic use	5 (9.4%, 3.1–20.6)	43 (18.2%, 13.5–23.7)	0.249
No. of stools in previous 24 h (median)	3.5 (IQR: 2–5)	5 (IQR: 3–7)	0.0324

^a^At least one of the following pathogens detected: *Campylobacter coli/jejuni, Salmonella* spp*., Shigella* spp*./E. coli enteroinvasive (EIEC), Yersinia enterocolitica, Toxin-producing Clostridium difficile*

**Fig. 2:**
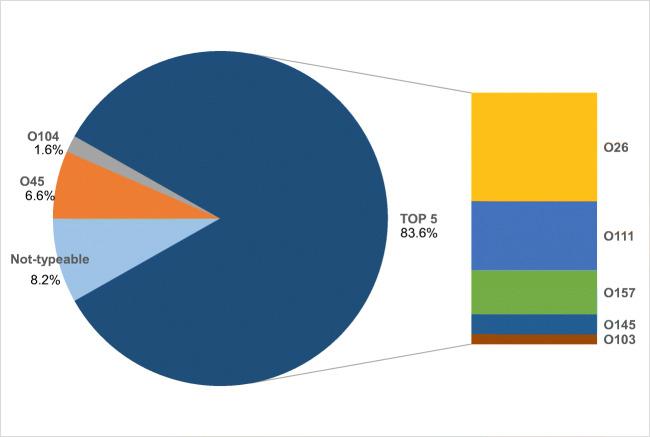
Distribution of STEC serogroups (%) in children with bloody diarrhea, Southern Italy 2018

Across all 53 STEC-positive children, 61 STEC serogroups were identified. In 8 of the 53 STEC-positive children (15.1%), two different serogroups were detected. Fifty-one of the 61 STEC serogroups detected (83.6%) belonged to the five most common STEC serogroups (O26, O111, O157, O145, and O103) (Fig. 2). The most common serogroups detected were O26 (*n* = 22, 36.1%) followed by O111 (*n* = 14, 23.0%). The O157 serogroup was identified nine times (14.8% of all detected serogroups), and the O145 serogroup was identified four times (6.6% of all identified serogroups). Other serogroups found were O45 (*n* = 4, 6.6%) and O104 (*n* = 1, 1.6%). In five cases, despite a positive result for the *stx1/stx2* gene in the real-time PCR test, a serogroup was not identified, probably because of a lack of analytical sensitivity in the test. In children younger than 2 years, the most common serogroup was O26 (*n* = 12/19, 63.1%). Thirty-one of 53 STEC-positive samples (58.5%) carried the *stx2* gene, 18 of whom were also positive for *stx1*. Four patients (all with STEC *stx1+/stx2+*) showed an evolution into HUS (7.5% of all STEC-positive children). All of these patients were female. The demographic and clinical characteristics of the four patients with a diagnosis of STEC-HUS are reported in Table 3. In two of these cases, neurological involvement was ascertained by clinical neurological evaluation, electrophysiological investigation (EEGs), and/or magnetic resonance imaging (MRI) of the brain. For these patients, eculizumab was administered after obtaining signed informed consent from the parents for “off-label” use of this drug [16]. All children with HUS showed a complete clinical recovery. In the patients with neurological involvement, one showed total regression of ischemic lesions in the follow-up through MRI (patient 2 in Table 3) while the other showed residual peripheral neuropathy with a complete functional recovery after rehabilitation (patient 4 in Table 3).

**Table 3:** Demographic and clinical characteristics of children with a diagnosis of STEC-HUS, Southern Italy 2018

Patient	Sex	Age	Onset of BD	Hospital admission	HUS diagnosis	Pathogen(s)	Clinical data	Neurological involvement	Therapies	Outcome
1	F	18 months	12/07/2018	14/07/2018	14/07/2018	STEC O26 (*stx1+/stx2+*) *C. difficile*	Oliguria, thrombocytopenia	No	Blood transfusion, plasma infusion, diuretics	Clinical recovery
2 (twin of patient 1)	F	18 months	17/07/2018	17/07/2018	19/07/2018	STEC O26 (*stx1+/stx2+*) *C. difficile*	Hemolysis, renal failure	Yes (alteration of consciousness, seizures. MRI^a^: hypoxic lesions of basal ganglia)	Blood transfusion, plasma infusion, hemodialysis, eculizumab	Clinical recovery
3	F	7 years	27/08/2018	28/08/2018	31/08/2018	STEC O145 (*stx1+/stx2+*)	Renal failure	No	Blood transfusion, plasma infusion	Clinical recovery
4	F	9 years	30/08/2018	30/08/2018	02/09/2018	STEC O111 (*stx1+/stx2+*)	Renal failure, hypotension	Yes (Decline in vision, alteration of consciousness, disorders of muscle tone. MRI^a^: ischemic lesions of corona radiata)	Hemodialysis, plasma infusion, eculizumab (antibiotic administered before hospital admission)	Inflammatory polyneuropathy with good functional recovery after rehabilitation

^a^*MRI* magnetic resonance imaging

## Discussion

To the best of our knowledge, this is the first report of the effects of a case management protocol based on laboratory surveillance of BD in a wide pediatric population in Italy. The results of a screening program for STEC detection in children with BD in the Lombardia region have been reported previously, although the clinical management of STEC-HUS cases was not described [7]. Moreover, Ardissino et al. focused only on bacterial coinfection in children with BD and did not report data on the prevalence of STEC infections during their 3-year study period. The surveillance protocol described in the present study represents the rapid response to an emerging public health threat in the Apulia region, Southern Italy, comprising an excess of pediatric HUS cases starting in 2013, with three deaths between 2017 and 2018 [13, 15].

It has been shown that 10–15% of patients with a STEC infection (mostly *E. coli* O157:H7) develop HUS after a median interval of 7 days from the onset of diarrhea [10, 17, 18]. Therefore, there is an opportunity to intervene to reduce the risk of developing HUS and/or avoiding sequelae such as neurological involvement through appropriate clinical management of the cases [16, 18]. Although there is a lack of specific therapy for STEC infection, when urine flow is preserved, early vigorous volume expansion reduces the risk of HUS and improves the short- and long-term outcomes [11, 12]. This formed the basis of our organizational model, which focused on prompt hospitalization of children with BD and early detection of STEC infection in the pediatric population [16, 18]. Moreover, re-evaluation of the laboratory parameters after 12/24 h, according to the protocol, is strongly recommended to monitor the possible evolution into HUS, which can occur rapidly [10].

During the study period, the mean annual estimated incidence of about 129 cases per 100,000 resident children is likely to be overinflated because the surveillance was carried out mainly in the summer months, when the risk of bacterial gastroenteritis is considered to be higher [19] and a high number of tourists populate the Apulia region, thus contributing to the case population but not the resident population. In about two-thirds of cases with BD, a bacterial pathogen was recognized. This is in accordance with previously reported data [20]. In our study, about 12% of cases with BD tested positive for STEC infection. A wide circulation of different STEC serogroups was observed, suggesting different sources of infection. More than 80% tested positive for the major STEC serogroups that are typically related to HUS [21]. The most common serogroup was O26 (36.1%), particularly in children aged less than 2 years (63.1%). These data were in accordance with the serogroup results reported for HUS cases in Italy and the Apulia region in particular over the last few years [13, 15, 22, 23]. In a 1-year prospective cohort study conducted in a single hospital in Missouri on 108 patients younger than 18 years with BD*,* McKee et al. found a STEC infection prevalence of 9% (10 cases) [20]. Although the overall prevalence of STEC infections in our study was comparable to that reported by McKee et al., they found that almost all their cases were due to serogroup O157, thus reflecting differences in STEC circulation between North America and Southern Italy [20].

In our study, fever and a higher number of loose stools were more common among positive non-STEC children than positive STEC children (p < 0.05). These data regarding fever were expected, since most STEC infections do not present with fever [24]. Moreover, there was no difference in the duration of symptoms at presentation between the two groups.

For correct clinical management of BD cases, early detection of STEC infection is crucial. Delays in specimen collection, delivery for laboratory evaluation, and in the STEC detection test itself may affect this aspect of case management [10]. Early diagnosis is a key point in the protocol used in the Apulia region. According to the protocol, the results of molecular tests were communicated to pediatric hospitalists within 24 h of the arrival of fecal samples. It is also important to avoid the incorrect use of antibiotics, since the administration of antibiotics is considered a risk factor for triggering HUS [25]. For this reason, another key point of the protocol is to avoid administration of antibiotics until etiological diagnosis. A relevant finding of this study was that antibiotic use was reported in 17% of BD patients at presentation. This suggests a misuse of antibiotics due to self-medication, or that primary care pediatricians may be not fully aware of this problem. Indeed, antibiotics for acute bacterial gastroenteritis should be administered only for specific pathogens or in defined clinical settings [26].

Of the 53 STEC children, four cases (7.5%) evolved into HUS despite a prompt STEC diagnosis, but all HUS cases had a favorable clinical outcome, including two cases that showed neurological involvement and were treated with eculizumab [16]. It should be noted that two of the four HUS cases (twins) were due to STEC O26, which may be particular virulent toward young children [15], and that in one of the other two non-O26 cases, an antibiotic was administered before hospitalization. The rate of evolution into HUS found in our study is lower than that reported in the literature [1, 2]. Overall, 15 HUS cases were reported in the Apulia region in 2018, with one of these patients not residing in Apulia (data from Regional Epidemiological Observatory, not published); four of these HUS cases were reported before the regional laboratory-based surveillance protocol was implemented, four were due to STEC infections identified by the protocol and that evolved into HUS, and five cases (registered between July and September) showed BD before hospitalization but escaped the enhanced surveillance and were admitted directly with a diagnosis of HUS. The remaining two cases did not show BD and were hospitalized with HUS because of a rapid evolution of the clinical picture.

The application of the regional BD surveillance protocol in Southern Italy suggests that if children presenting with BD to an ED are hospitalized, they can benefit from a prompt laboratory diagnosis and, in cases of STEC infection, from early volume expansion, which could reduce the risk of developing HUS and/or other complications. However, based on our data, two in four HUS cases showed neurological involvement, thus probably due to the strong virulence of the STEC serogroups O26 and O111, as previously reported [15, 16]. A more long-term application of the operating protocol here described would confirm this hypothesis and provide good and more reliable information.

This study has several limitations. First, the protocol has been active for a short time, and the implementation of the protocol may have been affected by a lack of dissemination among primary care pediatricians, public pediatric outpatient service pediatricians, and ED physicians, particularly during the first few months after the activation of the laboratory-based surveillance. This could also explain the occurrence of the five HUS cases in 2018 that lacked a previous diagnosis of STEC infection. Second, pediatricians from hospitals distant from the Regional Reference Laboratory may not have sent a fecal sample for all BD cases. Moreover, no information is available regarding the median time between the patients’ first access to care and their admission to hospital. Nonetheless, the data set used in this study is among the few available to have focused on hospitalization of all children with BD and, as such, can be used as a basis for further future studies. Continued pursuance of the regional protocol will allow a better understanding of whether laboratory-based surveillance of STEC infections and correct management of BD cases can reduce the impact of HUS in Southern Italy and will also add information about the epidemiology of STEC infections. Finally, the experience presented in this study demonstrates that increasing pediatricians’ awareness of the potential for STEC infections to evolve rapidly into HUS may prevent them from undervaluing cases of BD in children.

## Abbreviations

*BD*: Bloody diarrhea
*ED*: Emergency department
*EEGs*: Electrophysiological investigation
*HUS*: Hemolytic-uraemic syndrome
*MRI*: Magnetic resonance imaging
*STEC*: Shiga toxin-producing *Escherichia coli*
